# Evidence of the Cellular Senescence Stress Response in Mitotically Active Brain Cells—Implications for Cancer and Neurodegeneration

**DOI:** 10.3390/life11020153

**Published:** 2021-02-17

**Authors:** Gregory J. Gillispie, Eric Sah, Sudarshan Krishnamurthy, Mohamed Y. Ahmidouch, Bin Zhang, Miranda E. Orr

**Affiliations:** 1 Section of Gerontology and Geriatric Medicine, Department of Internal Medicine, Wake Forest School of Medicine, Winston-Salem, NC 27157, USA; ggillisp@wakehealth.edu (G.J.G.); esah@wakehealth.edu (E.S.); sukrishn@wakehealth.edu (S.K.); ahmimy17@wfu.edu (M.Y.A.); 2 Sticht Center for Healthy Aging and Alzheimer’s Prevention, Wake Forest School of Medicine, Winston-Salem, NC 27157, USA; 3 Bowman Gray Center for Medical Education, Wake Forest School of Medicine, Winston-Salem, NC 27101, USA; 4 Wake Forest University, Winston-Salem, NC 27109, USA; 5 Department of Genetics and Genomic Sciences, Department of Pharmacological Sciences, Mount Sinai Center for Transformative Disease Modeling, Icahn Institute for Data Science and Genomic Technology, Icahn School of Medicine at Mount Sinai, New York, NY 10029, USA; bin.zhang@mssm.edu; 6 Salisbury VA Medical Center, Salisbury, NC 28144, USA

**Keywords:** cellular senescence, Alzheimer’s disease, biology of aging, neurodegeneration, brain, geroscience, senolytics, tauopathy, cancer, stress response

## Abstract

Cellular stress responses influence cell fate decisions. Apoptosis and proliferation represent opposing reactions to cellular stress or damage and may influence distinct health outcomes. Clinical and epidemiological studies consistently report inverse comorbidities between age-associated neurodegenerative diseases and cancer. This review discusses how one particular stress response, cellular senescence, may contribute to this inverse correlation. In mitotically competent cells, senescence is favorable over uncontrolled proliferation, i.e., cancer. However, senescent cells notoriously secrete deleterious molecules that drive disease, dysfunction and degeneration in surrounding tissue. In recent years, senescent cells have emerged as unexpected mediators of neurodegenerative diseases. The present review uses pre-defined criteria to evaluate evidence of cellular senescence in mitotically competent brain cells, highlights the discovery of novel molecular regulators and discusses how this single cell fate decision impacts cancer and degeneration in the brain. We also underscore methodological considerations required to appropriately evaluate the cellular senescence stress response in the brain.

## 1. Introduction

The risk of both neurodegenerative disease and cancer increases with advanced age due to increased damage accumulation and decreased repair capabilities; yet the relative odds of developing one or the other are inversely correlated [1,2,3,4,5]. Molecular profiling studies have identified disrupted genes, proteins, and signaling pathways shared by neurodegenerative diseases and cancer, but in opposing directions. For example, p53 is upregulated in Alzheimer’s disease (AD), Parkinson’s disease, and Huntington’s disease, but downregulated in many cancers (reviewed [6]). Similarly, mutations of the Parkin gene *(PARK2)* have been shown to simultaneously contribute both to Parkinson’s disease and tumor suppression [7]. A recent study performed transcriptomic analyses of four different tissues from four different species at ages across their lifespan [8]. Across samples, the largest number of shared risk single nucleotide polymorphisms (SNPs) were in the genomic locus containing the long non-coding RNA ANRIL which modulates many cell cycle regulators including *CDKN2A/B* [9], which codes for p16^INK4A^ (hereon referred to as p16), one of the best characterized mediators of cellular senescence [10,11]. Notably, SNPs in this locus were identified in the brain, as well as other tissues analyzed [8]. These results point toward aberrant cell cycle, and in particular senescence, as a key age-associated molecular pathway worth further study.

Cellular senescence has emerged as a hallmark biological process that promotes aging (reviewed [12]). The pillars of aging, including cellular senescence, are highly interconnected and do not occur in isolation [13]. For example, epigenetic changes, telomere attrition, DNA damage and mitochondrial dysfunction all may induce cellular senescence, which then contributes to dysfunctional nutrient signaling and proteostasis. Consequences of cellular senescence include stem cell exhaustion and chronic inflammation. Thus, cellular senescence represents an intersection of aging hallmarks [13]. While best studied as an anti-cancer stress response, recent studies highlight its pro-degenerative role in AD and tauopathies [11,14,15]. As such, cellular senescence may contribute to the inverse correlation between the risk for developing neurodegeneration and that for cancer.

Bulk tissue analyses, while informative at a macroscopic level, may not capture important changes occurring in single cells. Senescent cell abundance increases with aging, but the relative contribution to a tissue is relatively low and may be missed in bulk analyses [16] (reviewed [11]). Several laboratories are using single cell technologies to assign cell type specificity to tissue-level observations [17], but to date these analyses have not included senescent cells in the brain. To maximize generalization and interpretation across studies, in this review we only evaluate studies which investigated cellular senescence with cell type specificity, and not bulk analyses. We specifically focus on mitotically competent brain cells; due to space considerations, the topic of postmitotic brain cellular senescence is reviewed in a separate manuscript (Sah et al., *Life*, in review). The present compilation provides evidence on conditions in which cellular senescence may benefit (anti-cancer) or negatively impact (neurodegeneration) brain health. In doing so, this review explores how the cellular senescence stress response may simultaneously distinguish and connect AD and cancer risk.

## 2. Identifying Senescent Brain Cells

**Figure 1:**
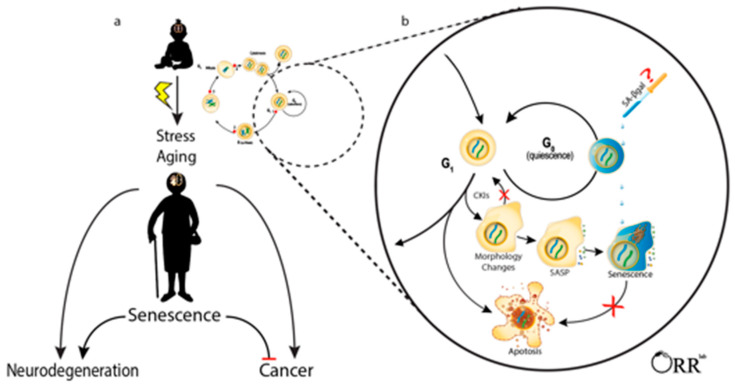
Cellular senescence in mitotically competent brain cells may impact risk for developing cancer and neurodegeneration. (**a**) The accumulation of cellular damage throughout the lifespan induces cell stress responses that impact cell fate decisions. Neurodegeneration increases with age and inversely correlates with risk of developing age-associated brain cancer. Cellular senescence may influence this clinical observation. (**b**) Mitotically competent brain cells may enter quiescence, a reversible G0 arrest. Damaged cells may undergo apoptosis, other cell death processes or senescence. In senescence, the cell permanently exits the cell cycle, upregulates cyclin-dependent kinase inhibitors (CKIs), exhibits morphological changes, acquires a senescence-associated secretory phenotype (SASP), and upregulates senescent cell anti-apoptotic pathways (SCAPs). Senescence-associated beta galactosidase (SA β-gal) staining can be used to identify some senescent cells but also labels some quiescent cells and requires careful methodological techniques and interpretations. For simplicity, senescence was illustrated at G1; however, cells may enter senescence arrest at G0 or G2 as well [64].

The identities of the parent cell type and upstream senescence-inducing stressors have consequences on the post-senescence phenotype [18,19]. The resulting heterogeneity has presented challenges for identifying, defining, and studying senescent cells in vivo and across disciplines. Senescent cells have been identified using various morphology markers; gene, protein, epigenetic, metabolic changes; and functional readouts and have been a subject of earlier reviews [20,21,22,23,24]. Transcriptomic analyses in particular show significant promise for identifying senescent cells. They have been utilized to study characteristics of senescence common between different cells-of-origin and modes of senescence induction and may help identify senescence markers which are more powerful than current traditional senescent markers such as p16 [25]. However, a specific combination of phenotypes defining cellular senescence currently does not exist [26]. Where biologists agree is that interpreting the senescence phenotype requires integrating various lines of distinct evidence placed in appropriate context [21]. This is especially true for postmitotic tissues such as the brain. We reviewed the literature using pre-defined senescence-defining criteria: proliferative/cell cycle arrest, apoptosis resistance, senescence-associated secretory phenotype, and senescence-associated β-galactosidase activity (Figure 1). As with measuring other biological processes, the interpretation of results requires integrating several lines of evidence. We explain the bases of these chosen criteria in this section.

### 2.1. Absence of Proliferation/Stable Cell Cycle Arrest

Replicative senescence, as observed by Hayflick and Moorhead [27], may be fundamentally restricted to mitotically competent cells. Brain cells which are susceptible to this fate include neural stem cells (NSCs), neural progenitor cells (NPCs), oligodendrocyte progenitor cells (OPCs), and microglia [28,29,30]. A subpopulation of astrocytes may be capable of cell division and trans-differentiating into neurons [31], however to what extent this happens in vivo and whether it occurs enough to induce replicative senescence in these rare cells is unclear. Of note, postmitotic cells may also undergo cellular senescence [32], including neurons [15,33,34,35]; a review of postmitotic senescent brain cells will be available in a separate manuscript (Sah et al., *Life*, in review). A wide range of methods have been validated to measure cell proliferation both in vitro and in vivo, including cell cycle specific markers, BrdU incorporation, ploidy, and the quantity and size of stem-cell containing non-adherent cultures (e.g., neurospheres or brain organoids). A reduction in proliferation at the tissue or cell population level (in situ tissue analyses or in vitro cell culture, respectively) may indicate that a portion of cells have undergone senescence or a general slowing of the cell cycle. To discern these differences and assign a senescence arrest, evaluating single cells is necessary.

Mechanistically, replicative senescence is achieved through intrinsically mediated (cell autonomous) telomere attrition [27] and has been the focus of several recent reviews [21,36,37]. Briefly, telomeres progressively shorten with age due to successive cell divisions and reduced telomerase activity. When chromosomes reach a critical truncated length, cells cease to replicate [36,37]. Average or relative telomere length can be measured using polymerase chain reaction (PCR), terminal restriction fragment (TRF) analysis, single-telomere length (STELA) analysis, and several different fluorescent in situ hybridization (FISH) methods [38]. Quantifying the shortest telomeres, as opposed to average length, may be more beneficial depending on the application and can be accomplished using the telomere shortest length assay (TeSLA) [38,39]. Similarly, telomerase activity can be quantified with a variety of measurement techniques including, most commonly, the PCR-based telomeric repeat amplification protocol (TRAP) [40]. A number of methods have also recently been developed, improving upon the convenience, throughput, sensitivity, and reliability of TRAP [41,42,43]. These methods provide objective readouts when evaluating potential mechanistic mediators of replicative senescence.

The most broadly accepted and universal phenotype among senescent cells is the change in cell fate that accompanies cell cycle arrest. This change in cell fate goes beyond a reduction in proliferation and is distinct from differentiation. While a reduction in cell proliferation can be reversible and attributable to many non-senescent causes, true senescence involves an irreversible cell cycle arrest. Senescent cells will not re-enter the cell cycle if signaled to do so, if the original stressor is removed, or even if reprogrammed with Yamanaka factors [44,45,46,47]. The stressors, pathways, and cell cycle inhibitors involved have been reviewed [45,48,49,50]. Many senescent cells accomplish cell cycle inhibition by upregulating p16 and/or p21^CIP1^ (hereon referred to as p21); many other cell cycle inhibitors have been reported as well [45]. Based on the prevalence of upregulated p16 across studies, reporter mice using the p16 promoter have been utilized for many of the in vivo experiments (reviewed [51]). A caveat to this in vivo work is its dependence on p16-mediated senescence, overlooking other pathways. Nonetheless, these results have guided the field to date. In summary, a stable cell cycle arrest indicative of cellular senescence includes the evaluation of cell proliferation; expression of cell cycle inhibitors, including p16, p21 and p19^INK4d^ (hereon referred to as p19); telomere attrition; and inability to re-enter the cell cycle when provided relevant stimuli. Additional morphological and functional changes accompanying senescence may include increased cell size; altered nuclei that are either enlarged (karyomegaly) or syncytia (multinucleated) [15,52,53].

### 2.2. Apoptosis Resistance

The enhanced survival of senescent cells depends on the activation of senescent cell anti-apoptotic pathways (SCAPs) [54,55]. The SCAP molecular pathways which have been identified thus far include BCL-2/BCL-XL, PI3K/AKT, p53/p21/serpines, dependence receptors/tyrosine kinases, HIF-1α and the unfolded protein response. The unfolded protein response in particular may be especially important for proteinopathies which cause many neurodegenerative dementing diseases. The senescence-associated phenotype can be identified by the expression of these SCAP pathways and/or by their resilience when exposed to apoptosis-inducing factors. Senescent cells often utilize multiple, redundant (although not all) SCAPs to survive in an otherwise toxic microenvironment. As such, targeting/evaluating multiple pathways is critical when identifying apoptosis resistance. Additionally, senescent cells should not express markers of apoptosis (i.e., TUNEL and caspase-3 negative).

### 2.3. Secretory Phenotye

The senescence-associated secretory phenotype (SASP) represents a deleterious effect of chronic senescent cell survival (reviewed [56,57]). Like other stressed cells, senescent cells secrete molecules to communicate to neighboring cells. These include chemokines and cytokines which signal to immune cells to clear them. Other secreted molecules include extracellular remodeling factors, exosomes, miRNAs, growth factors, and proteases that alter the environment and may induce senescence in other cells. The SASP is beneficial for tissue remodeling and wound healing (reviewed [21,58,59]). However, as senescent cells accumulate with age and pathology, the SASP contributes not only to local, temporary inflammation, but also chronic, systemic inflammation. This inflammation, which may have other contributors in addition to senescent cells, has been shown to be disadvantageous for a number of age-related diseases, including those in the brain. SASP factors differ across parent cell type and even within the same cell type exposed to different stressors. The phenotypic diversity of SASP factors represents a major challenge for developing a unifying profile and has been the subject of numerous reviews [56,57,58,59]. Moreover, how the SASP may change within a senescent cell over time remains unknown. Nonetheless, some of the common factors used to identify SASP in the studies reviewed here include IL-1α, IL-6, IL-1β, TNF-α, and CXCL1, CCL4, CCL6 and TGF-β.

### 2.4. Senescence-Associated β-Galactosidase

Senescent cells in vitro retain lysosomal β-galactosidase activity at pH 6.0, which is referred to as senescence-associated β-galactosidase (SA β-gal) activity [60]. The mechanisms surrounding SA β-gal activity are not fully elucidated, although studies have determined that *GLB1*, the gene encoding lysosomal β-D-galactosidase, is required [61]. This finding indicates an absence of a unique senescence-specific enzyme. Instead SA β-gal activity may reflect a change in lysosomal content or function. Toward this end, a recent study determined that lysosomes mediate whether cells remain quiescent or become senescent. As lysosomal function decreased, cells progressively lost the ability to reverse out of quiescence and thus became senescent [62]. However, the relation to SA β-gal staining was not investigated, so it remains unknown whether it coincides with the transition from deep quiescence to senescence. Notably, pharmacologically targeting lysosomal β-galactosidase clears senescent cells as evidenced by reduced expression of cell cycle inhibitor genes associated with senescence [63]. While these studies provide evidence for the utility of the SA β-gal assay to identify senescent cells, appropriately measuring SA β-gal activity requires careful methodological attention.

SA β-gal evaluation is a histological assay that requires enzymatic activity of GLB1. [61]. Due to this need for enzymatic activity, ideal experiments necessitate fresh frozen tissue, yet oftentimes the use of archived frozen or fixed tissues are reported in the literature. Similarly, it is imperative that the precise pH is reported and serial sections are processed at both pH 6.0 and pH 4.0 to discern between global differences in lysosomal activity from senescence-specific differences. Appropriate controls should include young, age-matched disease-free and/or untreated tissues that are processed side-by-side with the experimental samples.

Given that lysosomal function differences between quiescent and senescent cells are on a continuum and subtle [62], SA β-gal cannot be used as a single surrogate marker. Additionally, not all senescent cells acquire the phenotype. We emphasize the importance of considering the cellular phenotype in entirety when evaluating the senescent phenotype [21]; we used the methodological criteria described above to evaluate SA β-gal interpretations throughout the review.

### 2.5. Concluding Remarks on Identifying Senescent Cells

Evaluating cellular senescence requires both an appreciation for the phenotypic complexity of cellular senescence, expertise in the parent cell type and an understanding of how they respond to stress. Beyond the pre-defined criteria used in this review, additional markers may be applied to supplement the characterization of senescent cells. Morphological observations such as an enlarged, flattened morphology and a disrupted nuclear membrane and metabolic dysregulation such as increased ROS and mitochondria dysfunction are also commonly employed, among others [24]. In the following sections, we review studies reporting senescent cells of mitotically competent brain cells using the pre-defined criteria presented above and summarized in Figure 1. While reviewing the literature we evaluated the methods used to define senescence and how these phenotypes were placed in the context of brain cell biology.

## 3. Neuronal Precursor Cells

Brain maturation continues after birth for three months in mice [65] and 20 years in humans [66]. Even beyond that, the adult brain maintains populations of self-renewing, multipotent NSCs first identified in rats [67] and later in humans [68,69,70]. Adult neurogenesis occurs primarily in the subgranular zone (SGZ) in the hippocampal dentate gyrus (DG) and the subventricular zone (SVZ) around the lateral ventricles of the forebrain. In humans, the primary progenitor of the SVZ is a subpopulation of specialized, quiescent NSCs known as B cells which give rise to interneurons and OPCs. These are sometimes referred to as SVZ astrocytes due to their morphological structure and their co-expression of the glial fibrillary acidic protein (GFAP), Nestin and carbohydrate Lewis X (LeX) [71]. These specialized NSCs share some physical and molecular characteristics with typical astrocytes, including branched processes, intermediate filament bundles, cell bodies in the cytoplasm, and gap junctions [72]. Thus, precise phenotyping measures are required to identify neurogenic from non-neurogenic astrocytes. Some studies suggest that human NSCs populations persist and contribute to neurogenesis throughout adulthood [73,74,75,76,77,78,79,80]. Others have detected significant reductions during childhood, ranging from simply lower activity all the way to negligible or nonexistent in adults [81,82,83,84]. Thus, most studies reviewed in this section were performed in human cells in vitro or in mice.

In healthy adult mammalian tissue, NSCs exist primarily in a quiescent arrest [85]. During quiescence, cells lower their metabolic activity and cell division rate to minimize damage to DNA, proteins, and mitochondria which can lead to cancer, senescence, and exhaustion of the stem cell population [86,87]. Various environmental and behavioral activities have been shown to activate quiescent NSCs (e.g., exercise, sleep, learning) through diverse cell signaling pathways (e.g., neurotransmitters, Notch, neurotrophins, Wnt) [87]. Once activated, NSCs proliferate, migrate, and differentiate toward NPCs and OPCs and terminally differentiate into neurons, astrocytes, and oligodendrocytes [88,89]. Given that aberrant NSC activation may contribute to cancer, the ability for them to utilize the senescence stress response may benefit short-term health and survival.

The incidence of cancer, including in the brain, increases with age [90], and evidence suggests that NSCs are often the cells of origin for brain tumor formation [91,92]. Evolutionarily, the ability for proliferative cells to undergo senescence in response to cell damage provided an advantage over malignancy [93,94]. Some evidence suggests that patient survival rates are favorable when brain cancer cells become senescent [95]. Pilocytic astrocytoma is a slowly growing benign brain tumor derived from astrocytes and is the most common pediatric brain tumor. Genetic mutations in the proto-oncogene B-Raf gene, *BRAF*, cause pilocytic astrocytoma. Human fetal neurospheres transduced with a constitutively active form of *BRAF* were evaluated for senescence-like features. The mutation promoted colony formation and in early passages proliferation did not differ as assessed through BrdU incorporation. However, after five passages BrdU uptake was notably reduced in BRAF mutant cells compared to controls, and was shortly thereafter followed by proliferative arrest. The remaining cells appeared viable as per light microscopy and displayed a 35-fold increase in the percentage of cells with SA β-gal activity with elevated PAI-1 and p16 compared to controls [95]. The *BRAF* mutant NSCs displayed a progressive decrease in SOX2 expression that coincided with the increase in other senescence-associated protein upregulation, suggesting a loss of neural stemness as they progressed into senescence. Telomere length did not differ between NSCs indicating the senescence arrest was independent of telomere attrition. The research team confirmed elevated p16 in 86% (57/66) of patient-derived pilocytic astrocytoma tumors. Moreover, individuals whose tumors were negative for p16 (9 cases) had significantly shorter survival than p16 positive cases. Tumor suppressor TP53 (p53) was detected in 23% of pilocytic astrocytoma samples and significantly correlated with p16 in the cases that were scored. SA β-gal was also observed in >50% of primary tumor cells derived from patients (n = 2) at low passage number. Collectively, these data suggest that elevated p16, and presumably NSC senescence, is favorable for survival in patients with pilocytic astrocytoma.

Human NSCs may undergo cellular senescence in response to non-genetic carcinogens as well. For example, radiation decreased human NSC proliferation and caused cell cycle arrest as measured by BrdU uptake in vitro, acutely increased DNA damage repair (γH2AX levels returned to baseline by 2 hours) and apoptosis (PARP cleavage returned to baseline by 28 h) [96]. Cellular metabolism was also increased on a per cell basis, as measured by the XTT assay, and neuronal differentiation was impaired, suggesting the surviving cells were senescent rather than quiescent [96]. Irradiation also reduced proliferation of NSCs in the SGZ and SVZ of rats as measured by BrdU incorporation in vivo [97]. Neuronal differentiation was reduced 97% in vivo, but NSCs isolated from these rats were still able to differentiate towards neurons in vitro. Furthermore, non-irradiated NSCs also showed an 81% reduction in neuronal differentiation when transplanted into irradiated rats. Combined, these results suggest the effects of irradiation on NSCs may have been indirectly induced by the damaged microenvironment [97], thus highlighting the importance of healthy cells and environment; both of which are negatively impacted by senescence.

Canonical senescence phenotypes acquired by irradiated NSCs include cell cycle arrest and SA β-gal staining [98]. Unique features include an increased expression of astrocyte markers which has been observed in vitro [98] and in vivo [99]. This differs from astrocytes which downregulate GFAP when undergoing senescence [100]. Development of SASP has not reproducibly occurred in irradiated NSCs across studies. For example, Zou et al., did not detect SASP factors IL-6, IL-8, IL-α concomitant with cell cycle arrest and SA β-gal positive NSCs [98]. However, using a similar cell line, Schneider et al. reported radiation-induced NSC cell cycle arrest via p21 and p27^KIP^, and Rb-dephosphorylation [99]. These NSCs also showed an enlarged, flattened morphology and an increase in SA β-gal staining. In contrast to Zou et al., they found an increased secretion of several cytokines including IL-6 suggestive of a canonical senescence phenotype. Interestingly, irradiated NSCs still lost their markers of stemness (i.e., Nestin and SOX2) even when researchers inhibited the astrocytic differentiation pathway [99], indicating the reduced proliferation of these cells was not simply due to their differentiation into astrocytes. Together, these studies indicate that cell cycle arrest and SA β-gal positivity are not sufficient to label NSCs as senescent; however, these features combined with elevated p21 and p27^KIP^ and morphological changes co-occur with SASP production and more complete senescent phenotype.

Senescence phenotypes in NSCs have been observed in response to chemical carcinogens as well. Independently, Dong et al. [101] using NSCs derived from the SVZ of mice and Daniele et al. [102] using human NSCs derived from embryonic stem cells, observed senescence-like phenotypes after treatment with hydroxyurea (HU). HU is a ribonucleotide reductase inhibitor in the “antimetabolite” family of chemotherapies known to induce DNA damage. Both mouse and human NSCs showed senescent-like phenotypes including reduced proliferation via neurosphere formation, p21, and p16, increased DNA damage via p53 and γH2AX, and an increase in SA β-gal staining. A reduction in apoptosis was also found in NSCs derived from rats [101] and the proinflammatory transcription factor NF-κB was found in human NSCs [102]. Collectively, these in vitro studies suggest that NSCs develop features of senescence in response to HU, although further investigation into the irreversibility of this state, a more in-depth characterization of their resistance to apoptosis, and investigation of SASP production downstream of NF-κB activation would strengthen this assertion.

As so far, the literature indicates that senescence may protect NSCs from becoming cancerous in response to known carcinogens [103]. A long term effect of senescent cell accumulation is chronic inflammation and degeneration. The brain is especially susceptible to age-associated neurodegenerative diseases (reviewed [104]). For example, AD onset prior to the age of 50-years-old is exceptionally rare, with the exception of familial cases. In contrast, 10% of adults aged 65-years-old and roughly 40% over the age of 80-years old have an AD diagnosis. Notably, aberrant NSC behavior has been reported in patients with AD [105,106]. To explore whether AD pathology is a cause or effect of NSC senescence, we evaluated literature from postmortem human brain studies as well as mechanistic studies in vitro.

AD is a progressive neurodegenerative disease characterized by the presence of amyloid-β (Aβ) plaques and tau-containing neurofibrillary tangles [107]. Evidence from neurosphere assays suggests that Aβ_42_ [108] and tau [109] overexpression negatively impact NSC function through senescence-associated pathways. For example, He et al. [108] investigated senescence in response to Aβ_42_ oligomers in vitro and in vivo. Neurospheres derived from mouse hippocampi were exposed to various concentrations of Aβ_42_ for up to 5 days and assessed for proliferation, differentiation, SA β-gal, and toxicity. Apoptosis was increased with 10 µM Aβ_42_, but remained unchanged at lower concentrations. Aβ_42_ also reduced proliferation and increased p16 in a dose-dependent manner. In Aβ producing TgAPP/PS1 mice, the number of Nestin-positive NSCs double stained for p16 increased 1.3-fold and the number of SA β-gal positive cells in the dentate gyrus increased 1.7-fold. FPR2 (a G protein-coupled receptor which mediates the inflammatory response) and p38 MAPK (a stress response protein) were both implicated in the senescence induction of NSCs [108]. These in vivo data were largely driven by the use of p16 antibody staining and SA β-gal, both of which are somewhat unreliable due to lack of specificity (as discussed in [110] and [15], respectively). Nonetheless, the suggestive data are compelling for further investigation.

Tau protein accumulation is the most common intraneuronal pathology among neurodegenerative diseases [111,112]. We recently discovered that tau protein accumulation drives senescence in the brain [15], which was confirmed shortly thereafter by Bussian et al. [113]. Between the two independent studies, several senescent cell types were identified, but neither group looked at the role of tauopathy on NSC senescence. Nonetheless, evidence suggests that tau protein is critical for normal NSC behavior [109] and plays a role in abnormal activity in tauopathies [111]. We generated NSC-containing neurospheres from tau transgenic mice overexpressing either human frontotemporal dementia mutant tauP301L or human tau and compared phenotypes to those generated from wild type mice [109]. We found that mutant tauP301L overexpression resulted in abnormal proliferation and differentiation as indicated by MTT proliferation assays and filopodial/spine morphology measures, respectively. With repeated passaging, the non-adherent neurosphere culture proliferation rate significantly slowed and lost the ability to differentiate into brain cells. Instead, plating the Nestin-positive cells as adherent cultures revealed large, flattened cells with large cell somas containing numerous vacuoles and enlarged nuclei. These adherent cells remained in culture until finally discarding the culture plate after several weeks of attempts to expand the culture or differentiate into brain cells. While we did not examine cell cycle inhibitor expression or SA β-gal, the other defining features of cellular senescence were observed [109]. The Tau Consortium has generated at least 31 induced pluripotent stem cell (iPSCs) lines from 140 MAPT mutation/risk variant carriers and cognitively normal controls [114]. As the scientific community continues to utilize this resource, we anticipate more clarity surrounding the effects of aberrant tau on cellular senescence across brain cell types during early development and in disease.

Similarly, Voisin and colleagues [110] developed human iPSCs from a single patient with Huntington’s disease, a rare inherited neurodegenerative disease arising from mutations in the *HTT* gene. In this study, HTT CAG repeat-corrected (C116) cells from the same donors were used as a disease-free control. Using qRT-PCR and a gene ontology approach, they found expression of FOXO3 and its transcriptional targets to antagonize p16 expression. Furthermore, they identified p16 and related genes as key mediators of senescence in Huntington’s disease NSCs and medium spiny neurons. Cells derived from the Huntington’s disease patient additionally showed decreased proliferation and elevated SA β-gal staining in vitro [110]. These results suggest that Huntington’s disease mutations may negatively impact NSC function and perhaps contribute to disease through senescence-mediated pathogenesis; however, senescence phenotypes will need to be confirmed in postmortem human brain tissue from patients with Huntington’s disease to validate these intriguing preclinical findings.

Over 200 disease causing mutations to *POLG*, a subunit of the mitochondrial DNA repair protein polymerase gamma (polγ), have been identified—many of which primarily affect the nervous systems. Using fibroblasts from two patients carrying *POLG* mutations, Liang et al. [115] generated iPSCs and then differentiated them into NSCs. While the iPSCs recapitulated some known *POLG* mutation phenotypes, the NSCs especially showed characteristic signs of mitochondrial dysfunction, including reduced energy production, abnormal mitochondrial volume, respiratory chain complex 1 loss, and increased ROS. Other evidence include upregulated mitochondrial uncoupler protein 2 (UCP2, which maintains mitochondrial membrane potential) and reduced phosphorylated Sirtuin 1 (SIRT1, a NAD-dependent protein deacetylase) [115]. In addition to mitochondrial dysfunction, these NSCs showed increased levels of SA β-gal staining and p16 expression suggestive of senescence. While the study did demonstrate SA β-gal staining and potentially a reduction in cell proliferation via p16, their characterization of senescence did not include the cell’s resistance to apoptosis or a SASP. Instead the focus of the study was primarily on mitochondrial dysfunction given its strong link with *POLG* mutation disorders. Together these studies highlight the on-going research and approaches utilized by the neuroscience field to understand mechanisms surrounding age-associated neurodegenerative diseases. Cellular senescence is emerging as a potentially significant cell stress pathway worth continued investigation.

While the above studies provided examples of the effects of carcinogens, genetic or pathological variants on NSC function, other studies have investigated sporadic, age-associated phenotypes. Tissues accumulate senescent cells with advanced chronological age. Gao et al. [116] isolated SVZ neurospheres in vitro from 23-month-old mice and compared them to 3-week-old counterparts; an age where developmental neurogenesis has completed but the brain is still maturing. Neurosphere formation, proliferation, and neuronal differentiation were significantly lower in aged NSCs. Further, aged NSCs showed increased p16 expression and shortened telomeres via qRT-PCR. SA β-gal staining, apoptosis, and SASP were not investigated. These changes were partially alleviated by flavonoids of the plant Ribes meyeriy, [116] suggesting the drug’s anti-aging and potentially senotherapeutic effects.

Using the senescence accelerated (SAMP8) mouse model at 2, 6, and 12 months of age, Hu et al. [117] showed an age-related increase in NSC senescent phenotypes. NSCs, labelled via Sox2 and either Nestin or GFAP, were found to decrease with age in the hippocampus. Furthermore, SA β-gal staining in the hippocampus increased at 6 months and again at 12 months. The percentage of senescent NSCs, labelled via Sox2 and p16, also increased with age. Isolated NSCs from these mice were less able to form neurospheres or differentiate into neurons. DNA damage-associated cell cycle arrest was evidenced by elevated γH2AX, p53, p16, and p21. Exogenously applied embryonic stem cell extracellular vesicles reduced many of these senescence-associated outcomes both in SAMP8 mice and in vitro. RNA sequencing analyses revealed that *Myt1*, a regulator of neurogenesis, was downregulated with passaging and age and upregulated with the extracellular vesicle treatment. Consistent with these observations, *Myt1* knockout mice developed many of the same senescence-associated phenotypes and were resistant to the beneficial effects of the extracellular vesicles [117]. These data suggest that *Myt1* may be a novel regulator of NSC senescence and that extracellular vesicles from embryonic stem cells have potential to treat age-related neurodegeneration.

A recent study by Xiao et al. [118] investigated senescence phenotypes in aged hypothalamic NSCs. Neurospheres derived from 18-month-old wild type mice were fewer, smaller, and had lower proliferation than those from 3-month-old mice. From these experiments, they identified the long non-coding RNA Hnscr as playing a role in NSC senescence. *Hnscr* null mice displayed similarities to wild type counterparts at young ages, but accelerated aging at 18 months, including reduced NSC proliferation and increased SA β-gal staining. Similar, but less striking, results were seen with viral suppression of *Hnscr* specifically in hypothalamus NSCs. RNA sequencing and bioinformatic approaches revealed cell senescence, apoptosis and inflammatory responses among the altered pathways. In particular, p16 was upregulated as confirmed by qRT-PCR. Lastly, wild type mice treated with Theaflavin 3-Gallate, an Hnscr mimetic, had lower NSC senescence compared to vehicle treated controls. Outcome measures included SA β-gal staining and RNAseq data consistent with cell cycle arrest, apoptosis resistance, and a SASP suggest a *bona fide* senescence arrest. Collectively their data highlight Hnscr as a critical mediator of hypothalamic NSC senescence. Future studies are needed to determine whether this broadly applies to other NSC populations or SCs in general. Nonetheless, this study highlights a significant advance in senescence biology by evaluating brain cell populations.

Overall, these studies indicate that NSCs acquire senescence-like phenotypes in response to carcinogens and stressors known to promote cancer or neurodegenerative disease. Given the inverse relationship between the risk for developing AD and cancer, it is tempting to speculate that senescence may protect against cancer but promote neurodegeneration. However, further research is needed to draw more conclusive inferences. Through reviewing the literature, we identified important areas for clarifying “senescence” in NSCs. One is the difference between quiescence and senescence. Given that cell cycle arrest is a defining feature of both, the permanency of the arrest needs to be experimentally determined. A gold standard assay to confirm the cell cycle activity in NSCs is their ability to divide and give rise to multipotent clones when provided with cytokine growth factors in the culture, e.g., the neurosphere assay. While useful to identify proliferative arrest ex vivo; cell cycling is not easily discernable in postmortem tissue, especially since similar molecular pathways are used for both quiescent and senescence arrest. For example, the transcription factor Bmi-1 regulates NSC self-renewal by suppressing p16 and p19 [119,120,121]. p16 regulates whether DG NSCs exit quiescence and undergo neurogenesis in response to running [122] and genetically removing p16 lessens the age-associated neurogenesis decline in the SVZ, but not DG [123]. *Cdkn1a*, which codes for the cell cycle inhibitor p21, expression is required to appropriately regulate NSC proliferation/quiescence during early and middle age [124], and in response to ischemia [125]. These studies highlight the physiological roles of commonly used markers of cellular senescence (i.e., Bmi-1, p16, p19 and p21) in NSC maintenance and behavior in an age, stress and region-specific manner. Thus, their differential expression in histological tissues may represent a snapshot of regulated NSC quiescence, making it difficult to discriminate from senescence. Distinguishing between quiescent and senescent NSCs in particular remains a challenging roadblock to the field.

Other distinguishing markers can help differentiate senescence from quiescence or other causes of decreased neurogenesis. For example, senescent stem cells remain metabolically active whereas a reduction in metabolism is one of the key characteristics of quiescence [87,126,127]. While little is known about the secretome of quiescent cells, limited evidence suggests a potential overlap with the SASP including IL-1α, IL-6, IL-8, CCL4, and CCL6 [128,129]. Apoptosis resistance, to our knowledge, has not been investigated as a marker of NSC senescence. Nonetheless, NSCs naturally display a general resistance to apoptosis [130,131]. Whether or not these pathways change with age or in response to other stressors could be used to help distinguish between senescence and quiescence. A recent study identified a gradual entrance into senescence from quiescence regulated by lysosomal function [62]. While this may complicate the discernment between quiescence and senescence, it suggests that lysosomal content and activity may help decipher these cell states.

### Concluding Remarks

In summary, true senescence may have been present in some of these studies, but most have only partially characterized the phenotype. Other studies have taken for granted senescence-related phenomena which occur in other cell types, but have not yet been demonstrated in NSCs. The Hayflick Limit and the SASP, for example, both represent opportunities for future experimentation in NSCs. In addition, while many studies have relied heavily upon SA β-gal staining, few have adhered to proper protocols (see Section 2.4.). Nonetheless, mechanistic studies utilizing brain NSCs are advancing our understanding of the molecular regulators of cellular senescence, including neurodegenerative disease-associated tau [109], *Foxo3* [110], *Myt1* [117], and lncRNA Hnscr [118]. Whether or not they are cell type specific remains unknown, but they highlight the utility and importance of evaluating senescence in brain cells. Future studies will be needed for more in-depth characterization of senescent NSCs, their effects in brain pathology, and interventions specifically targeting this cell population.

## 4. Oligodendrocyte Precursor Cells

OPCs form 5–8% of all cells in the adult brain [132]. Developmentally, OPCs arise from multiple progenitor cell pools in the developing spinal cord and forebrain (reviewed [133,134]). Postnatally, OPCs are predominantly found in the SVZ and migrate to white matter regions, where they proliferate and differentiate into oligodendrocytes and astrocytes [134,135]. OPC multipotency provides an opportunity for malignant gliomas [136] including oligodendroglial [137] and astrocytic tumors [138]. Physiologically, OPCs differentiate into oligodendrocytes throughout life and contribute to myelin turnover [139,140,141,142], adaptive myelination [141,143,144] and regenerative processes following demyelinating insults [132,145]. The ability of OPCs to respond appropriately to myelin maintenance or stress signals determines whether they positively impact myelination [132,139,140,141,142] or potentially divide uncontrollably and become cancerous [136,137,138]. While cellular senescence protects against the latter, evolutionary pressure for the senescence stress response did not account for the long lifespans of modern day humans. OPC senescence, thus, may contribute to neurodegenerative phenotypes in later life. Evidence for the tradeoff between OPC senescence, cancer and neurodegeneration is presented in this section.

When grown in optimal culture conditions, OPCs challenge the Hayflick limit and replicate indefinitely [146]. Prolonged culture results in elevated expression of many cell cycle inhibitors (Cip/Kip proteins: p21, p27 and p57, and INK4s: p18 and p19) in the absence of cell cycle arrest. This non-senescence profile may be attributed to significant upregulation of positive cell cycle regulators Cdk2, Cdk4 and cyclin D1, D3 and E [146]. These experiments suggest that a healthy brain environment could provide OPCs an opportunity for indefinite replication (e.g., could be beneficial for brain maintenance but contribute to cancer). However, suboptimal culture conditions including prolonged exposure to 15% fetal bovine serum, contact inhibition due to overgrowth/confluency, and low dose genotoxic drugs all produced senescence-like phenotypes. Culture shock conditions resulted in proliferative arrest, including a failure to incorporate BrdU, and flattened morphology [146]. Notably, elevated p16 was not detected in any culture conditions, but viral-mediated overexpression of p16 strongly inhibited BrdU incorporation [146]. While these results indicate that p16 activation can cause OPC proliferative arrest, OPCs do not innately upregulate p16 in response to many known senescence-inducing stressors. Additionally, p21 is required for OPC differentiation to oligodendrocytes [147], which presents challenges for interpreting OPC differentiation from senescence. Thus, two of the most commonly used markers of senescence, p16 and p19, require additional considerations and methodologies and highlight the importance of including measures of DNA damage response pathways, resistance to apoptosis, and the SASP when evaluating OPC senescence.

Age-associated deficits in remyelination often co-occur with a lack of OPCs in the lesion site (e.g., poor recruitment) and/or delayed or failed OPC differentiation. The successful execution of recruitment and differentiation requires a complex interaction between the OPCs and their environment. Thus, poor remyelination may reflect impaired OPCs and/or suboptimal environmental cues. Cell autonomous deficits in OPCs are inferred by increased DNA damage, upregulated *Cdkn2a*, and reduced cellular respiration in OPCs isolated from 20-24 month-old mice compared to 2–3 month-old mice [148]. Given the increase in DNA damage, as evidenced by single cell comet assays, poor OPC proliferation and migration may protect against the development of malignant gliomas. In this way, a senescence response may be beneficial over tumorigenicity. Similarly cultured OPCs from aged mice were less proliferative as shown by decreased BrdU incorporation and cell cycle arrest [149]. Examining whether these cells are resistant to apoptosis and identifying SASP factors associated with the cellular environment would validate the senescence phenotype in OPCs, and provide much needed information regarding whether they acquire a toxic SASP or remain benignly arrested.

OPCs are present abundantly in grey and white matter, and are believed to survey their microenvironment, migrate to lesioned areas for remyelination through repopulation of oligodendrocytes, and respond to inflammatory cues [132,150]. Although the non-progenitor characteristics of OPCs are less understood, they respond to CNS injury, ischemia, and neurodegeneration, along with microglia [150,151]. Depletion of the endogenous OPC pool, reduced migration of OPCs in a pathological environment, and lack of differentiation of OPCs lead to failure of remyelination in brain lesions, suggesting a critical role for OPCs in neurodegenerative diseases [134]. Interestingly, in post-mortem human brain tissue of patients with AD, elevated levels of p21 were observed in OPCs surrounding Aβ plaques, but not in regions devoid of pathology [152]. The authors reported that >80% of large Aβ plaques (>50 μm) contained cells that co-expressed Olig2 and p21. Interestingly, brain samples from non-demented controls or with mild cognitive impairment (MCI) contained few Olig2 or NG2 positive cells regardless of p21 or p16. This observation suggests that the Aβ plaque environment induces OPCs to proliferate, migrate and/or hone to the plaque environment. Given that p21 is a marker of OPC differentiation, one possible explanation for elevated p21 expressing OPCs near plaques is that they are differentiating into oligodendrocytes, which may be delayed by the plaque environment as described with aging [153]. Similarly, multiple sclerosis brain lesions more often contain OPCs that have failed to differentiate rather than a lack of OPCs [154,155,156]. In rodent models of these conditions, increasing the number of OPCs does not improve remyelination [157], presumably due to the unfavorable environment. Thus, it is tempting to speculate that the increase in p21 expressing Olig2 cells near Aβ plaques [152] represents a proper OPC response to proliferate and migrate to the lesion, but the unfavorable plaque environment has delayed or prevented their differentiation into mature oligodendrocytes. In this way, identifying the Aβ plaque-associated molecule(s) that impair OPC differentiation could provide an opportunity for therapeutic intervention. Across studies, additional markers are needed to distinguish whether p21 expressing OPCs are actively differentiating into oligodendrocytes (albeit slowly) or senescent.

Mouse models of Aβ plaque accumulation support the notion that OPCs hone to the plaque environment. The 3xTg-AD mouse model develops Aβ and tau pathology characteristic of patients with AD [158]. Hypertrophic OPCs were observed surrounding and infiltrating Aβ plaques in brains of 3xTgAD mice, which again suggests that OPCs hone to regions of Aβ pathology [159]. Similarly, TgAPP/PS1, mimicked the findings of human brain whereby increased *Cdkn2a* mRNA expression, SA β-gal, and IL-6 levels (SASP factor) were found in OPCs surrounding Aβ plaques to suggest that extracellular protein accumulation may negatively impact OPC function [152]. A novel ZsGreen senescence marker utilizing the p16 promoter was crossed to TgAPP/PS1 mice. The TgAPP/PS1 × ZsGreen mice displayed elevated reporter expression compared to wild type ZsGreen mice indicating significant upregulation of p16 [152]. Treatment with senolytics, Dasatinib and Quercetin [54], alleviated senescence phenotypes, reduced the presence of IL-1β, IL-6 and TNF-α (SASP factors), ameliorated cognitive deficits, and reduced neuroinflammation in OPCs of TgAPP/PS1 mice [152]. Although senescent cells were cleared with administration of senolytics, Aβ load remained unchanged in these mice [152], which may suggest that senescent cells alters cognition independent of Aβ. Moreover, these results may suggest that senolytics cleared the negative regulator of OPC differentiation thus allowing them to develop into mature oligodendrocytes. In this case, other senescent cells may be negatively impacting OPC function through their SASP. Indeed other senescent cell types have been identified in AD including neurons with neurofibrillary tangle pathology [15]. Interestingly, enhanced OPC function has been reported in mice expressing mutant human tau that drives senescence in many other brain cell types including neurons and microglia [15,113,160]. The authors conclude that damaged axons, possibly from senescent tau-containing neurons, promotes OPC differentiation. It is tempting to speculate that neuronal SASP may be responsible for this altered OPC behavior. Indeed, our analyses of transcriptomic differences between neurons with or without NFTs indicate that NFT-bearing senescent neurons [161] differentially express growth factors that may influence OPC behavior.

OPC senescence has been suggested to contribute to disease progression in rodent models of progressive multiple sclerosis (PMS) [162]. High-mobility group box 1 (HMGB1) was identified as a component of the secretome of senescent NPCs [162]. Moreover, HMGB1 induced OPC senescence, as defined by upregulated gene expression of *Cdkn2a*, *Mmp-2*, and *Igfbp2*, and impaired the ability to OPCs to differentiate into oligodendrocytes [162]. The authors interpret that treatment of NPCs with rapamycin, an mTOR inhibitor, reversed the senescence phenotype as evidenced by a decrease in *Cdkn2a* mRNA and protein expression, and decreased SA β-gal intensity [162]. OPCs cultured in media isolated from these rapamycin-treated NPCs were found to differentiate into oligodendrocytes at a higher rate than OPCs cultured in PMS NPC media without rapamycin treatment, further suggesting a role played by senescent NPC environment in successful differentiation of OPCs [162]. Given that senescent cells are stably arrested, we question whether the NPCs in the PMS model are instead in deep quiescence and perhaps not fully senescent [62]. Nonetheless, the beneficial effects of rapamycin on OPC function warrant further investigation for demyelinating diseases.

### Concluding Remarks

OPC multipotency offers the brain regenerative capacity throughout life, but at the cost of cancer development. OPCs migrate to lesions, proliferate, and repopulate the area with oligodendrocytes, in an effort to remyelinate and repair. Due to their ability to replicate indefinitely in culture, cellular senescence may play an important protective anti-cancer role in vivo. While OPC senescence-associated phenotypes have been identified in vitro, the in vivo data require further investigation. In particular, the most compelling study in AD used p21 as a marker of OPC senescence [152]. However, given that p21 upregulation is required for OPC differentiation, we cannot discern if these OPCs are experiencing delayed differentiation or if they became senescent and can no longer differentiate. Many of the studies suggest that OPCs hone to sites of injury but fail to differentiate; determining whether these cells represent senescent OPCs requires further investigation. Though several studies have demonstrated senescence-like phenotypes in OPCs, we could not find evidence for SASP or SCAPs. These areas are of primary interest as they will inform on whether future therapeutic approaches should focus on the use of senolytics to treat OPC dysfunction.

## 5. Microglia

Microglia are known as the primary immune cell of the central nervous system, consisting of 0.5–16.6% of all brain cells in humans [163]. While neurons, astrocytes, and oligodendrocytes originate from the neural tube, microglia are derived from yolk-sac macrophage precursors and migrate to the brain before the blood-brain barrier forms [164,165]. Microglia mostly display postmitotic phenotypes with an estimated turnover rate of 0.08% per day in healthy human cortex with ^14^C birth dating indicating an average age of 4.2 years [166]. However, they readily proliferate locally in response to injuries [167,168,169]. Recent studies have also shown that there are latent microglia progenitors that can proliferate and differentiate into microglia in the microglia-depleted brain [170].

Microglia serve a dual function of maintaining the health of the central nervous system and acting as brain macrophages with immune surveillance by monitoring their cellular environment and promoting repair. For example, they remove dying neurons [171], protein aggregates such as Aβ [172] and tau [173], and tissue debris, as well as support synaptic pruning [172]. Through their release of anti-inflammatory cytokines and trophic factors such as IL-4, IL-10, IL-13, TGF-β, they contribute to neuronal and oligodendrocyte survival [174,175]. However, they also can be activated to secrete pro-inflammatory cytokines, TNF-α, IL-1β, IL-6, MIP-1α, reactive oxygen species, and nitric oxide [176,177]. Opposite to anti-tumorigenic pro-inflammatory cytokines, anti-inflammatory cytokines and trophic factor secretion by microglia are known to be pro-tumorigenic as they promote an immunosuppression of the tumor microenvironment, tissue repair, and angiogenesis that enable tumor invasion and survival [178]. Brain cancers such as gliomas are infiltrated with glioma associated microglia (GAM) that are recruited and reprogrammed by glioma cells [179,180]. These microglia increase glioma growth due to their decreased tumor sensing and immune response, while releasing mitogens and invasion promoting factors [181,182]. Given the role of microglia in neuroprotective and immune-modulatory functions, aberrant microglial function has also been linked to neurological diseases such as AD and PD [165].

Numerous studies have reported that microglia can become senescent and/or dystrophic. In the microglia literature, “dystrophic” and “senescent” are often used interchangeably [183]. However, we note that this is distinct from the cellular senescence criteria we use to evaluate the literature. Specifically, dystrophic microglia show morphological changes such as altered cytoplasmic structures and iron overload, but do not necessarily display the other criteria (i.e., shortened telomeres, cell-cycle arrest, lack of proliferation, resistance to apoptosis, and SASP). Activated microglia secrete proinflammatory cytokines and reactive oxygen species similar to senescent microglia. The key difference between activated and senescent microglia is that senescent microglia are unable to proliferate. Here we review microglia senescence, focusing on the pre-defined phenotypes outlined in Section 2.

Activated microglia are those in a hypersensitive state with exaggerated inflammatory responses. Activating stimuli include natural aging, infections, and other systemic inflammatory episodes [184]. For example, aging microglia display impaired neuroprotective abilities including low motility, reduced phagocytic capacity, and the secretion of pro-inflammatory molecules and reactive oxygen species. Repeated intraperitoneal injection of lipopolysaccharide (LPS) induces similar effects of systemic inflammation both in vivo and in vitro. In response to these stressors, activated microglia secrete pro-inflammatory molecules and reactive oxygen species. To identify different types of activated microglia including the traditionally defined M1 (pro-inflammatory) and M2 (anti-inflammatory) states, mRNA expression or protein levels of inflammatory markers such as *TNF-α*, *IL-1β*, and *TLR2*, and histological analysis of morphology markers such as *Iba-1*, *CD68*, and *CD11b* have been commonly examined [177,185,186,187]. Single-cell RNA sequencing has recently been used identify a more broadly defined activation response microglia (ARM)/disease-associated microglia (DAM) to examine specific microglial activation gene signatures including *APOE*, *CST3*, and *CD74* [188]. In contrast, various distinctive measures have been used as a biomarker for microglia senescence including detection of senescence-associated heterochromatin foci (SAHF), accumulation of lipofuscin, SA β-gal activity, secretion of matrix metalloproteinase-1 (MMP-1), nitrotyrosine, and other pro-inflammatory molecules, loss of lamin B1 expression, and upregulated p53, p21, and p16 markers [164,189,190,191,192]. Altered autophagy and impaired mitochondrial functions were also reported [176,192]. Thus, it is important to not only examine the morphology and secretion of pro-inflammatory molecules of microglia, but also to examine other markers of senescence to distinguish senescent microglia from activated microglia.

Studies in vitro have revealed that microglia can acquire various senescence-like phenotypes. Primary microglia obtained from an amyotrophic lateral sclerosis (ALS) rat model expressing SOD1^G93A^ developed flat morphology, SA β-gal, and increased expressions of p16, p53, and MMP-1 at 12 days in vitro compared to 2 days in vitro. [191]. A similar experiment looked at microglia cells isolated from neonatal mice and compared the cultured cells at 2, 10, and 16 days in vitro [193]. In comparison to culture day 2, day 16 cells revealed altered morphology, increased matrix metalloproteinase (MMP-2) activation, NF-κB activation, miR-146a expression, SA β-gal activity and decreased autophagy and expression of Toll-like receptor TLR-2 and TLR-4 [193]. Cell culture studies have demonstrated that telomere shortening contributed to microglia senescence. Reduced expression of murine *Tert* and telomere-associated genes were observed in activated primary murine microglia from C57Bl6 mice using mRNA isolation and qRT-PCR [194]. The transcriptomic changes associated with telomerase activity is believed to contribute to microglia senescence as reduced *Tert* mRNA expression is also observed in both ischemia and AD-like pathology in vivo [194]. Experiments in vitro with the BV2 microglia cell line have shown that LPS stimuli repeated 6 times every 48 hours induces acute neuroinflammation, drives heterochromatic foci, increases p53 expression, and increases SA β-gal staining [195]. Thus, chronic inflammation has been suggested as an intrinsic and extrinsic inducer of microglia senescent phenotypes [193,195].

Interestingly, human microglia isolated from Braak stage III or greater AD neuropathology also revealed a higher quantity of microglia with short telomeres compared to that of non-demented individuals using the same assays [196], which provides evidence that microglia from human AD brain with advanced tau neuropathology may be susceptible to cellular senescence. Notably, tau accumulation induces a microglia senescence-like phenotype in transgenic mice [113]. Isolated microglia from 6-month-old TgPS19 transgenic mice that express high levels of mutant human tauP301S showed increased expression of cell cycle regulators p16, p19, and p21, and pro-inflammatory IL-6 compared to control mice through fluorescence-activated cell sorting (FACS) and qRT-PCR analysis [113]. Although this study did not evaluate telomere length, together with the human brain data [196] and other studies on tau-associated senescence [15], the data suggest that neuronal tau pathology may induce a senescence phenotype in microglia.

A relationship between telomere length and senescence has been demonstrated with natural aging as well. Microglia isolated from 30-month-old mice displayed significantly shorter telomeres via telomere flow-FISH and decreased telomerase activity via TRAP compared to the younger controls [196]. Compared to natural aging microglia, LPS injection can generate a model for microglial senescence relatively quickly. Mice from several different studies display strong pro-inflammatory responses to LPS injection, such as large increases in *Tnfα*, Il-6, and Il-1*β* expression in both a neurodegenerative mouse model of ME7 prion diseases and in middle-aged mice [197,198]. While these pro-inflammatory markers are consistent with SASP, this study [197] did not address other features of senescence such as loss of proliferation, DNA damage, aberrant expression of cell cycle inhibitors, and resistance to apoptosis. Notably, not all markers of senescence fully translate to in vivo studies. An absence of p21/p53 pathway activation, lack of telomere shortening, and increased number of Ki-67 markers were reported for microglia isolated from 24-month-old mouse brains [199]. The authors concluded that the aged microglia may be dysfunctional, but that they do not display a senescent profile [199]. This striking discrepancy between widely accepted in vitro senescent markers and in vivo studies requires better approaches for understanding the differences between induced senescence in vitro and aging in vivo. Furthermore, despite how inflammation can trigger a senescence-like profile in microglial cells, the discrepancy in results between the natural aging and primed microglia studies should perhaps urge researchers to reexamine primed microglia as a model for cellular senescence beyond their pro-inflammatory responses.

### Concluding Remarks

Altogether, these studies verified microglial senescence-like phenotypes through observations of morphology [191,193,199], DNA damage [194,196,199,200], lack of proliferation [199], cell-cycle arrest machinery [113,191,195,199], SA β-gal [191,193,195,199], and pro-inflammatory SASP [113,184,193,199]. It is important to note that microglial senescence is complex and heterogeneous. In order to highlight microglial cells as senescent and not simply dysfunctional, we conclude that telomere shortening, activation of cell-cycle arrest machinery, lack of proliferation markers, resistance to apoptosis, and pro-inflammatory SASP markers are crucial to verify microglial senescent state. The commonly used SA β-gal assay may provide additional insight into microglia lysosomal activity, but cannot be used as a single surrogate to identify senescent cells. In this regard, of the reviewed studies, the study by Stojiljkovic et al. [199] met the predefined criteria outlined in the introduction. As the microglia senescence field moves forward, we recognize that clarity on definitions is needed. Oftentimes, dystrophic and senescent are used interchangeably, but do not imply biological cellular senescence as described in this review. Moreover, activated microglia display many characteristics of senescence. Careful evaluation of the full senescence phenotype is needed to make the distinction.

## 6. Summary

The accumulation of senescent cells has been reported across tissues in model organisms. Several recent studies have implicated senescent cell accumulation with neurodegenerative diseases [15,34,113,152,201]. Given that age increases both the incidence of neurodegenerative diseases and senescent cell accumulation, determining directionality and causality in the human brain remains unclear [14]. The articles reviewed in this manuscript support the possibility that the cellular senescence stress response may prevent the development of brain cancer. In this way, cells that undergo senescence may evade cancer, but drive neurodegeneration in later life. Thus, the cellular senescence response may contribute to the predisposition of individuals towards either cancer or neurodegeneration. This relationship, however, is complicated by a multitude of factors which have been identified to concurrently increase the risk of neurodegeneration, senescence and cancer. Senescence itself may also increase the incidence of cancer via the SASP [202].

Several concurrent characteristics are needed to positively identify senescent cells. These include proliferative arrest, a permanent change in cell fate, the SASP, a resistance to apoptosis, and possible staining with SA β-gal. Evaluating these criteria in vivo and in situ presents challenges, especially in the human brain. As such, most studies evaluated in this literature review did not measure or meet all of our predefined criteria for defining cellular senescence. While most senescent phenotypes were observed in the brain cell types reviewed, they were rarely observed simultaneously in a single study. By only partially characterizing the phenotype, the results leave room for alternative explanations beyond senescence. A reduction in proliferation and increase in p16 or p21 could indicate quiescent, not senescent NSCs. In particular, whether senescent NSCs exhibit SCAPs has not yet been investigated and their secretory phenotypes have been rarely characterized. Features associated with the senescent phenotype of OPCs are relatively less known. While it has been established that the surrounding environment strongly influence OPCs, further study of the in vivo senescent phenotype is required to reveal SASP or SCAPs associated with OPCs. Current literature utilizes p21 as a biomarker for the senescent phenotype. However, the upregulation of p21 in the differentiation process of OPCs confounds the use of this marker in senescence. In microglia, the SASP was a primary method for identifying senescent cells, but activated microglia also secrete pro-inflammatory molecules and reactive oxygen species. In general, a lack of other methodologies were used to verify that microglia cells were senescent and not simply activated in these studies. Future studies should further verify a resistance to apoptosis in senescent microglia and differentiate between activated, dystrophic, and senescent microglia via proliferative markers.

Staining with SA β-gal is perhaps the most prominent method for identifying senescent brain cells. However, the mechanistic link between SA β-gal staining and senescence remains unclear. The strong staining of quiescent postmitotic neurons additionally calls into question the utility of the method especially in the brain [15,152]. Additionally, many studies discussed in this review used archived tissue and did not utilize proper methods including insufficient controls, replicates, and poor reporting of pH. Lastly, the difficulties associated with dual labeling with SA β-gal makes it difficult to discern which cell type is being observed in vivo, meaning most evidence of cell-specific SA β-gal staining comes from in vitro studies.

Great advances have been made in the past few years regarding the presence of senescent cells in vivo. The use of rodent models carrying reporter transgenes have provided conclusive evidence that senescent cells accumulate with age across tissues. As scientists work to translate these findings to human tissue, there is a need to develop techniques and methodologies to identify, track and study senescent cells at the single cell resolution similar to what has been accomplished in mice. With the advancement of single cell transcriptomics and high-resolution digital profiling, we are on the precipice of developing similar data for human tissues [17,26]. Such data coupled with advanced systems biology approaches [203,204,205,206] provide an opportunity to identify robust multianalyte patterns across cells simultaneously and at the single cell resolution. As emphasized throughout this review, no single marker can identify senescent cells. Multianalyte transcriptomic and proteomic data therefore will provide the opportunity to confidently identify senescent cells. Directionality, magnitude and concurrence of senescence-associated pathways will be important in data interpretation. For example, many of the cell types here caution against the utility of using gold standard p16 and p21, especially in isolation, given their critical importance for brain cell function (i.e., NSC quiescence, OPC differentiation, microglial activation). However, a co-occurrence of upregulated cell cycle inhibitors, SCAPs and SASP with concomitant reduction in cell proliferation, differentiation and lysosomal pathways would provide stronger evidence that a cell has entered senescence. We expect comprehensive bioinformatic analyses of these multianalyte measures will also reveal cell-specific SCAPs and SASP with detailed signaling circuits that can be used for drug development. Given that clinical trials for the treatment of AD are underway (NCT04063124 and NCT04685590), results from high resolution studies will be critical in advancing this therapeutic approach.

In conclusion, though additional work is needed to confidently identify, profile and study senescent cells in the brain, this area of research holds great promise. We have highlighted several studies which, while focused on a specific cell type, have broader impacts for senescence in the rest of the body. Novel mechanisms regulating senescence have been explored in brain cells [110,117,118] and senescence in the brain has been shown to have major effects on the entire organism [207]. Future studies focused on senescence in the brain will continue to advance the fields of biology of aging, neuroscience and cancer; and potentially lead to interventions that broadly impact human health and longevity.

## Data Availability

Not applicable.
